# Taurine Stabilizing Effect on Lysozyme

**DOI:** 10.3390/life12010133

**Published:** 2022-01-17

**Authors:** Leonardo Mastrella, Paolo Moretti, Silvia Pieraccini, Simona Magi, Silvia Piccirillo, Maria Grazia Ortore

**Affiliations:** 1 Department of Life and Environmental Sciences, Marche Polytechnic University, I-60131 Ancona, Italy; leonardo.mastrella@uni.lu (L.M.); paolo.moretti@univpm.it (P.M.); 2 Department of Chemistry “Giacomo Ciamician”, University of Bologna, I-40126 Bologna, Italy; 3 Department of Biomedical Sciences and Public Health, Marche Polytechnic University, I-60200 Ancona, Italy; s.magi@univpm.it (S.M.); s.piccirillo@staff.univpm.it (S.P.)

**Keywords:** Small Angle X-ray Scattering, Circular Dichroism, spectroscopy, amyloid, taurine, protein solvation, lysozyme

## Abstract

Taurine is an important organic osmolyte in mammalian cells, and it weakens inflammation and oxidative stress mediated injuries in some diseases. Recently, taurine has been demonstrated to play a therapeutic role against neurodegenerative disorders, although its parallel involvement in several biochemical mechanisms makes not clear taurine specific role in these diseases. Furthermore, the stabilizing effect of this molecule in terms of protein stability is known, but not deeply investigated. In this work we explore by Circular Dichroism the stabilizing impact of taurine in lysozyme thermal denaturation and its influence in lysozyme aggregation into amyloid fibrils. Taurine even at low concentration modifies protein-protein interactions in lysozyme native state, as revealed by Small Angle X-ray Scattering experiments, and alters the amyloid aggregation pattern without completely inhibiting it, as confirmed by UV/Vis spectroscopy with Congo Red and by Atomic Force Microscopy. Evaluation of the cytotoxicities of the amyloid fibrils grown in presence or in absence of taurine is investigated on SH-SY5Y neuroblastoma cells.

## 1. Introduction

Folding of proteins into their soluble and functional form occurs through a delicate and multi-step pathway of conformational adjustments: the fully native state is achieved when all the hydrophobic side chains find their close packing into the central core and water is excluded [1]. Reversible folding-unfolding transformations have a crucial role in regulating biological activities and organisms developed various methods for limiting misfolding and its effects [2,3], however either in vivo or in vitro proteins can be destabilized and self-aggregate into insoluble deposits known as amyloid fibrils [1]. At the molecular level, in these conditions hydrophobic segments normally buried become exposed to the water solvent and non-native intermolecular self-assembly becomes predominant. The resulting fibrillar assemblies exhibit a well organised cross- β -sheet structure originated by H-bonded β -strands running perpendicular to the fibril axis [4].

In recent years protein misfolding and consequent amyloid deposition has become the subject of extensive research mainly due to connection with highly debilitating and increasingly prevalent human diseases [5,6]. These pathologic conditions, generally called amyloidosis, include neurodegenerative disorders such as Alzheimer’s disease (AD), Parkinson’s disease, Corea of Hungtington, Type II Diabetes Mellitus, and systemic diseases like immunoglobulin light chain, transthyretin and dialysis-related amyloidosis. More than thirty human proteins ranging from globular proteins to unstructured peptide molecules are associated to amyloid disorders (e.g., amyloid β peptide to AD, α -synuclein to Parkinson’s Disease, transthyretin for familial transthyretin amyloidosis), but it is now widely accepted that the ability to form amyloid aggregates is a rather general property of polypeptide chains [5,7]. Amyloid fibrils derived from different polypeptides show indeed common properties and structure regardless of the aminoacidic sequence, and this is one of the reasons why well-known proteins like lysozyme can be considered a good model for amyloid research, too [8]. Many studies have been devoted to investigate the fibril formation mechanism [9] and to identify natural [10,11] or synthetic compounds effective as antiamyloid agents [12,13,14]. Among naturally occurring molecules, osmolytes are increasingly investigated as potential therapeutic tools against amyloidogenic disorders [15]. These highly soluble species including amino acids and their derivatives, polyols and methylamines, are accumulated intracellularly in response to denaturing events (fluctuations in salinity, cold and heat stresses), and are well known to protect proteins and their functions acting as stabilizing or destabilizing mediators [16]. Different hypothetical models have been reported for describing the mechanism of protein-osmolyte interaction, referring to either a direct interaction or an indirect effect based on the action on solvent molecules [17,18,19,20,21]. On this latter issue, the exclusion mechanism driven by unfavourable interactions between the peptide backbone and the osmolyte is often considered responsible for the preferential hydration of the protein domain and its consequent stabilization. In addition, it has been seen that several osmolytes are stabilizing or destabilizing depending on their concentration and the chemico-physical parameters of the environment such as temperature and pH [22].

Taurine (2-aminoethanesulfonic acid) is a stabilizing osmolyte classified as a compatible solute in terms of both protein stability and function [23]. The molecule is a β -amino acid, containing the amino (pKa = 9.06) and the sulphonate (pKa = 1.50) functional groups, found abundant in mammalian cells and involved in multiple physiological functions including thermoregulation, anti-inflammation, antioxidation, calcium homeostasis, retina and central nervous system (CNS) development [24]. This molecule has been reported to help in membrane stabilization and neuroprotection, and its depletion has been correlated to an increased risk of several dysfunctions including Parkinson’s [25] and AD [26]. Very recently taurine has been increasingly suggested as therapeutic agent for AD treatment [27]. In 2017 Jang et al. have shown that this amino acid can help the recovering of cognitive functions in AD model mice [28]. In 2019 taurine was found to reduce the passive avoidance memory impairment induced by AD in scopolamine treated rats [29]. Although taurine shows versatile pharmacological role to ameliorate several neurological disorders, the molecular basis of its action against these disorders is not deeply understood, mainly because taurine action is related to many different biological processes. Indeed taurine has been suggested to promote the genesis, survival and growth of neurons in the hippocampus [30], to inactivate microglia-dependent inflammation in the CNS [31], and to be involved in several molecular mechanisms recently reviewed [32]. As osmolyte, taurine intracellular level increases in response to an increase in osmotic stress, while it reduces stress due to hypo-osmosis. Both of them are crucial mechanisms to defend cells from extreme stretching in response to osmotic inequalities. It modifies different osmolyte levels such as the Na ${}^{+}$ level, which has numerous dynamic functions in the cellular environment, including transport and membrane potential. Unlike for other osmolytes, in vitro investigation of taurine’s effects on protein aggregation and thermal stability are rarely reported in literature and, to our knowledge, just a few papers deal with its anti-amyloidogenic effect evidenced on human serum albumin [33], glucagon [34] and tau and amyloid- β -peptide [35]. Experimental data on the taurine stabilizing effect against lysozyme thermal denaturation were collected at pH 6.8 by UV measurements [36], while DSC calorimetry provided information in unbuffered water solution either on lysozyme or ubiquitin for taurine concentrations ≤ 400 mM [37]. Different mechanisms have been proposed for explaining taurine effects on protein stability, ranging from the preferential exclusion of the osmolyte from the water-lysozyme interface [36] to an ordering effect working on water molecules that are so restrained from attacking the hydrophobic core of Chimotripsin Inhibitor 2 [38]. More recently, the hypothesis of a direct interaction of taurine with the protein surface has emerged [37,39].

For providing additional insights into the stabilizing properties of taurine and/or taurine-protein interaction we carried out a parallel investigation of the effects of increasing taurine concentrations on thermal denaturation and amyloid fibrillation, by using lysozyme as a well-estabilished model protein [8,40,41,42]. A variety of conditions have been already developed in vitro to induce fibrillation in hen lysozyme, and mutations in human lysozyme can cause accumulation of large quantities of amyloid in liver, kidney, and other regions of gastrointestinal tract. It follows that understanding the mechanism of lysozyme aggregation could probably have therapeutic implications for the treatment of systemic non neuropathic amyloidosis [8]. Also lysozyme is a stable and compact protein: this fact makes possible a comparison between the stabilizing effect against thermal denaturation and the inhibiting effect against amyloid aggregation, which has been found for other osmolytes like trehalose. On the other side, considering a disordered protein like amyloid β peptide or α -synuclein, which have a well-known link to specific neurodegenerative diseases, would prevent us from performing investigation testing the protective effect of the osmolyte against unfolding.

In our experiments, the examined lysozyme solutions were buffered at acidic pH—in accordance with the fibrillation protocol here adopted—and Small Angle X-ray Scattering (SAXS), Circular Dichroism (CD), and UV spectroscopy experiments were performed at the same protein concentration of 3 g L ${}^{-1}$ , with increasing taurine concentrations. In particular, even a taurine concentration higher than 400 mM was explored by CD. Lysozyme is a glycosidase composed of 129 amino acids (isoelectric point about 11.2) stabilized by four disulphide bonds. At pH 2.3 lysozyme molecules possess about 18 elementary positive charges. The protein shows a globular native shape, with a α + β conformation, that under suitable stressful conditions (high temperature, low pH, high ionic strength, mechanical agitation, presence of cosolvents or cosolutes) is widely known to undergo amyloid fibrillation. The thermostability of the native structure under the influence of different taurine concentrations was monitored by UV/CD spectroscopy, while Congo Red binding assay was used to evaluate, for the different examined conditions, the inhibitory ability of the molecule on amyloid fibrillation by following the kinetic of the aggregation process. CD analysis was additionally performed for revealing the conformational evolution of lysozyme during amyloidogenesis either in the absence or in the presence of a selected amount of taurine. AFM imaging provided a morphological characterisation of the aggregated species. In order to evaluate the possibility that taurine influences protein-protein interactions even in conditions not affected by stress (high temperature and/or agitation), SAXS experiments were performed on native lysozyme solutions at increasing taurine contents exclusively until 100 mM, which can be considered more interesting if compared to those found in physiological conditions [43]. Finally, cytotoxicity of the amyloid fibrils grown in presence and in absence of taurine was investigated on SH-SY5Y neuroblastoma cells.

## 2. Materials and Methods

### 2.1. Sample Preparation

Lysozyme from lyophilized white eggs purchased from Sigma-Aldrich (St. Louis, MO, USA), was mainly used at a concentration of 3 g L ${}^{-1}$ (10 g L ${}^{-1}$ for SAXS), dissolved at pH 2.3 as in previous protocols [44,45]. Samples were prepared for in-solution SAXS experiments on native conditions, thermal denaturation and fibrillation experiments. In all cases, a 6 g L ${}^{-1}$ lysozyme solution (or 20 g L ${}^{-1}$ for SAXS analysis) was prepared in acidic buffer (pH 2.3) and maintained at 4 ${}^{\circ }$ C overnight. The day after the solution was diluted with the proper buffer containing the right amount of taurine and/or NaCl before performing experiments (final concentrations of taurine from 25 to 600 mM, and final concentration of NaCl from 5 to 50 mM). Fibrillation was induced, after the night at 4 ${}^{\circ }$ C, by incubation at 65 ${}^{\circ }$ C, with gentle agitation, according to literature [44]. Taurine was purchased from Sigma-Aldrich and added in proper amounts to lysozyme solutions.

### 2.2. Small Angle X-ray Scattering

SAXS data were collected at the Austrian beamline of Elettra Synchrotron in Trieste, Italy [46]. Measurements were carried out at 20 ${}^{\circ }$ C. Scattering patterns were recorded using the Pilatus3 1 M detector system (Dectris, Switzerland), and the transmitted X-ray beam was measured by a photodiode mounted on the beamstop. The radial average of the 2D detector images provided the scattering intensity as a function of the magnitude of the scattering vector *Q* defined by the equation Q=4πsinθ/λ , with 2θ being the scattering angle and λ equal to 1.54 Å the X-ray wavelength. The incident and transmitted intensities were measured. Data were corrected for sample transmission and fluctuations of the primary beam. The individual scattering patterns arise from the average of all images of each sample; the averaged respective backgrounds, treated in the same way, were subtracted from the average of all images. The sample stage was injected by the μ -Drop sample changer recently designed and developed in the Austrian beamline [47]. This μ -Drop system offers some advantages over a capillary based set-up, between them the small sample volume used (15 μ L). Each measurement was performed on at least 5 injections of sample volumes of 15 μ L, and was carried out 4 times for 10 s for each injections, hence each spectrum is the average of 20 spectra. To minimize the effects of radiation damage in the protein sample, each SAXS acquisition was followed by 3 s of deadtime. After SAXS data comparison, we averaged all the measured scattering data corresponding to the same nominal sample. Buffer measurements were always performed before and after sample measurements. Protein solutions and buffers were measured at the same conditions concerning temperature and exposure time. Lysozyme solutions at concentrations equal to 3 g L ${}^{-1}$ and to 10 g L ${}^{-1}$ had been measured, in order to check protein native conformation, at lower concentration, and to explore protein-protein interactions, at higher concentration. We did not observe radiation damage on samples presented in this study. SAXS data analysis was performed by GENFIT software package [48].

### 2.3. UV-Visible Spectrophotometry

UV-Visible absorption spectra were recorded at regular time intervals on lysozyme solutions in presence of Congo Red (CR) (Sigma-Aldrich, St. Louis, MO, USA) and provided the kinetics of fibrillar aggregation of lysozyme, either in the absence or in the presence of different amounts of taurine and/or NaCl. Due to its high affinity for β -sheet conformations of fibrillated proteins [49], CR dye is commonly used as a diagnostic tool for amyloidogenesis. In fact, tissues with amyloid aggregates become of red colour if stained with CR alkaline solutions. It means that CR bonding with β -sheet structures induces a red-shifting of CR absorption maximum from ≃500 nm to ≃540 nm. CR solution was prepared by its dissolution in ethanol (80% *w*/*w*) and addition of NaCl up to saturation. The obtained solution was then saturated with the dye powder and filtered with Millipore filters of pore size 0.2 μ m to remove possible aggregates as in previous experiments [45,49].

Due to characteristic pH-dependent changes in the CR absorption spectra, lysozyme solutions were brought at pH ≃ 7, combining 50 μ L of the selected protein sample (pH ≃ 2) with 50 μ L of the CR solution, and 400 μ L of the buffer brought at pH 11.5 with NaOH.

CR amount was selected by performing preliminary spectrophotometric measurements on several samples obtained with different volumes of CR solutions added to lysozyme solutions. We carried out a semi-quantitative analysis providing the fraction of β structures formed in solution: the ratio between absorption intensity at λ = 538 nm and at λ = 505 nm was calculated for each sample. Because the two wavelengths correspond to CR absorption maximum observed when it is associated with β structures, and to CR absorption peak when the dye is in its free state in solution, this ratio can be considered proportional to β structures in solution [45]. For each measurement a quartz cuvette with a 1 cm pathlength was used.

For each result presented in this study, at least nine measurements were made, of which only the mean values are reported.

### 2.4. Circular Dichroism

CD measurements were performed with a Jasco J-715 spectropolarimeter (Jasco, Tokyo, Japan), by using a circular quartz cell of 0.1 mm path-length. Spectra were recorded from 260 to 190 nm at 50 nm/min by taking the average of five scans. Each spectrum was corrected for the baseline by subtracting the spectral contribution of the buffer solution. Data are expressed in units of millidegree. To monitor amyloid fibrillation spectra, measurements were recorded at room temperature, after samples reached thermal equilibrium. On the other side, variable temperature measurements were carried out for thermal unfolding investigations. In these latter case, experiments temperature was controlled by a Neslab RTE-111 circulator thermostat (temperature stability ± 0.5 ${}^{\circ }$ C) (Newington, NH, USA), and a cell equipped with a thermostating jacket was used.

### 2.5. AFM

Atomic Force Microscopy measurements were carried out on an AIST-NT Scanning Probe Microscopy (Horiba Scientific, Kyoto, Japan). Images were carried out in non-contact mode with a pyramidal silicon tip, with radius of 8 nm. Samples were obtained from the end points of the fibrillation processes investigated by UV-Vis spectroscopy, diluting from the original concentration by 1:1000. The dilution ratio has been optimized for the first set of samples and then repeated at fixed value for every sample condition. 5 microliters of the diluted solution were deposited on a freshly cleaved mica surface and then leave to incubate for 20 min. After incubation, the sample was rinsed with milli-Q water, and dried with nitrogen blow down. All images were acquired with a resolution of 512 × 512 pixels with a scan rate of 1 Hz and were analyzed with the Gwyddion software (version 2.58). For each sample condition at least 5 images have been acquired.

### 2.6. Cell Culture and Treatments

The human neuroblastoma cell line SH-SY5Y was obtained from American Type Culture Collection (CRL-2266). SH-SY5Y cells were cultured as a monolayer and grown in polystyrene dishes (100 mm diameter) in Dulbecco’s Modified Eagle’s Medium (DMEM; Corning, New York, NY, USA) supplemented with 10% fetal bovine serum (FBS), 100 U/mL penicillin, and 100 μ g/mL streptomycin (Corning, New York, NY, USA) and were maintained in a humidified incubator at 37 ${}^{\circ }$ C in a 5% CO ${}_{2}$ atmosphere [50]. To assess the effect of lysozyme fibrils on their survival, SH-SY5Y neuroblastoma cells were plated on 12-well plates and, after 24 h, the different types of fibrils where added at a final concentration of 20 μ M. After 72 h, the cells were harvested for further analysis.

### 2.7. Cell Viability Assay

The cytotoxicity of fibrils was determined by using the 3-(3,4-dimethylthiazol-2-yl)-2,5-diphenyltetrazolium bromide (MTT) (Sigma-Aldrich) assay. The MTT assay measures cell viability by assessing the ability of mitochondrial dehydrogenases (the succinate-tetrazolium reductase system) (Sigma-Aldrich) to metabolize the yellow tetrazolium salt MTT to purple insoluble crystals of formazan [51]. Briefly, at the end of the experimental procedure, the cells were incubated with 0.5 mL of MTT solution (0.5 g L ${}^{-1}$ in PBS) in the dark at 37 ${}^{\circ }$ C and a 5% CO ${}_{2}$ atmosphere, in a humidified incubator. After 1 h, the cells were washed with PBS, and the produced formazan crystals were dissolved in 0.5 mL of DMSO [50,52,53]. A decrease in mitochondrial activity resulted in a reduction in the amount of formazan produced and therefore in the absorbance value. Absorbance was read at a wavelength of 540 nm using a Victor Multilabel Counter plate reader (Perkin Elmer, Waltham, MA, USA). The results were expressed as percentages of the control value.

All the data are expressed as the mean ± standard error of the mean (S.E.M.). The statistical analysis was performed by using GraphPad Prism 5 software (San Diego, CA, USA). One-way ANOVA analysis followed by Dunnett’s post hoc test were used to calculate the differences in mean values. *p* < 0.05 was considered to be a statistically significant difference.

## 3. Results

### 3.1. Native Interactions

**Figure 1:**
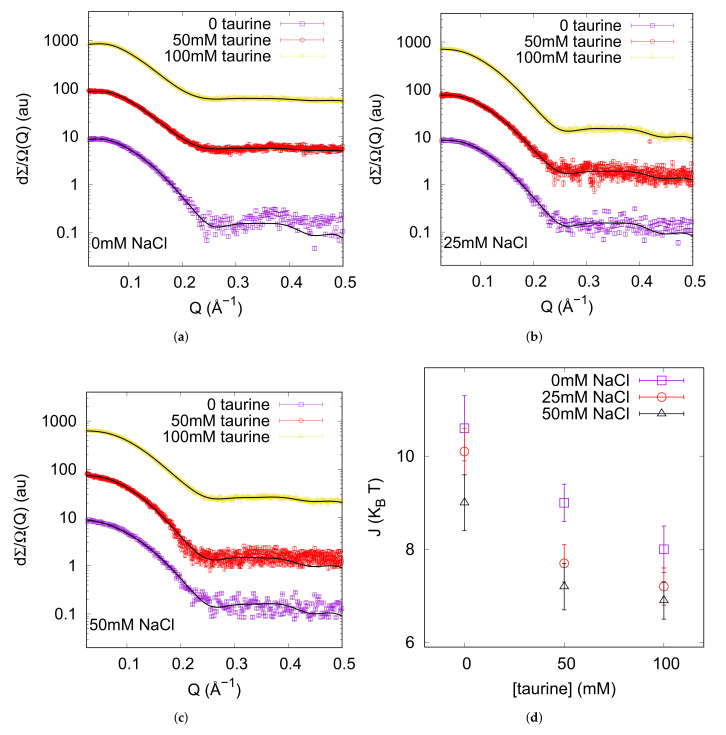
From top to down: in the three panels (**a**–**c**) SAXS experimental curves corresponding to native lysozyme at c = 10 g L ${}^{-1}$ , in presence of increasing contents of NaCl, from 0 to 50 mM as in the legend, and of taurine, from 0 to 100 mM as in the legend, are reported. Continuous lines represent the theoretical fitting, performed according to the model described in Section 3. Figure (**d**) reports the depth of the attractive potential between lysozyme molecules resulting from SAXS data global fit analysis as a function of taurine and NaCl contents.

We performed SAXS experiments on native lysozyme solutions in order to investigate the role of taurine in genuine conditions. Lysozyme solutions at low concentration (≃3 g L ${}^{-1}$ ), the same protein concentration used for thermal unfolding monitored by CD and amyloid aggregation monitored by CD and UV spectroscopy, were used to evidence possible effects on lysozyme hydration due to the presence of taurine in solution. However, from 0 up to 100 mM taurine SAXS profiles of lysozyme solutions completely overlap (data not shown), suggesting no solvation change due to the presence of taurine detectable by SAXS. Note that a study on protein solvation shell composition would require SANS experiments with contrast variation method [54], which is below the aims of our present work. Hence, protein structure and its solvation features can be considered constant under the conditions investigated by SAXS in this study. On the other side, we performed SAXS experiments at higher protein concentration in order to determine feasible effects of taurine in protein-protein interactions. Data reported in Figure S1 in the Supplementary Materials clearly evidence the presence of a structure factor at the higher investigated protein concentration. Also, slight but detectable differences can be evidenced in the structure factor responsible for protein-protein interactions, as Figure 1 reports. The macroscopical differential cross section resulting from SAXS experiments is the product of a form factor, describing protein three-dimensional structure, and of a structure factor which takes into account protein-protein interactions [55]. While SAXS data corresponding to lysozyme at a concentration of 3 g L ${}^{-1}$ reported in Figure S1 can be fitted just by the protein form factor, SAXS curves obtained from a 10 g L ${}^{-1}$ lysozyme solution need to be fitted by the product of a form factor and a structure factor. The form factor was calculated from 6LYZ pdb entry [56] according to the protocol described in [57], while the structure factor arises from the sum of an hard sphere potential, a screened coulombic potential and an attractive Yukawa term, as described in [54]. We analysed the whole set of SAXS curves at lysozyme concentration c= 10 g L ${}^{-1}$ in presence of 0, 50, and 100 mM taurine and of 0, 25, 50 mM NaCl added to protein solutions, by a global fitting approach via GENFIT software [48]. Protein charge was considered a common parameter and it could vary in the 10% in respect to literature values [58]. The ionic strength was constrained in the 5% error in respect to its nominal value and it was a single curve parameter. The dielectric permittivity of taurine solutions was obtained from the supplementary information of [59]: it increases at increasing taurine concentration, up to ≃83 in our experimental conditions. After fitting trials, the range of the Yukawa attractive potential was fixed to 5.8 Å for all the investigated conditions, while the depth of the attractive potential *J* was a single curve fitting parameter. Last, the ratio between the electron density of protein solvation shell $\rho_{s}$ and the bulk one $\rho_{0}$ was a single curve parameter, in order to consider possible influence of denser water and/or of taurine in the solvation shell. However, the ratio $\frac{\rho_{s}}{\rho_{0}}$ is comprised between 1.06 and 1.10 in all the investigated conditions, without any trend, in agreement with literature results [60].

The main outcome of SAXS data analysis are shown in Figure 1d. The depth of the attractive potential slightly decreases at increasing taurine concentration in solution, and this behaviour is maintained at increasing ionic strenght. *J* decreases from ≃10 $k_{B}T$ to ≃7 $k_{B}T$ in absence of NaCl, and this reduction of about 25% of its original value when taurine addition is coupled with ionic strenght is almost maintained. These results prompt that the attraction term between lysozyme proteins in solution decreases at increasing taurine content in the mixture, in agreement with the trend evidenced in the results obtained with lysozyme at normal pH by Julius et al. [59]. Absolute values of the attractive parameters cannot be simply compared, because buffer conditions were not equal and because in our approach we fixed the attractive range to restrict the number of fitting parameters. Note that quite at the opposite the increase of the dielectric permittivity as a function of taurine addition, determines a reduction of protein-protein coulombic repulsion. According to Julius et al. [59], taurine effect against protein-protein attraction is enhanced at higher taurine concentration, up to 750 mM, when the attractive potential depth halves. On the other side, TMAO, another osmolyte, produces the opposite effect, increasing the attractive potential. Remember that although most of the stabilizing osmolytes enhance the water structure and can be classified as water structure-maker solutes, there are some exceptions in this group, such as taurine [61]. Taurine has been demonstrated to be a water structure breaker [37] which produces in its surrounding two diverse populations of affected water: weakly bonded water molecules around the sulfonate group, and strongly bonded ones around amino group. In a certain sense, NH ${}_{3}^{+}$ group enhances water structure, while SO ${}_{3}^{-}$ weakens it. Without doubts, taurine affects water network, and consequently protein-protein interactions in solution.

### 3.2. Thermal Unfolding

In order to explore the taurine effects on protein stability we performed thermal unfolding experiments on different selected solutions and used CD spectroscopy to monitor the conformational change of the protein. Analysis of data provided the midpoint transition temperature ( $T_{m}$ ), i.e., an estimation of the stability of the folded state. UV/CD signals arising in the far-UV spectral region are associated to the n- π * (210–220 nm) and the π - π * (190 nm) electronic transitions of the peptide chromophore, and characteristic CD band-shapes are detected for the different secondary structures [62].

**Figure 2:**
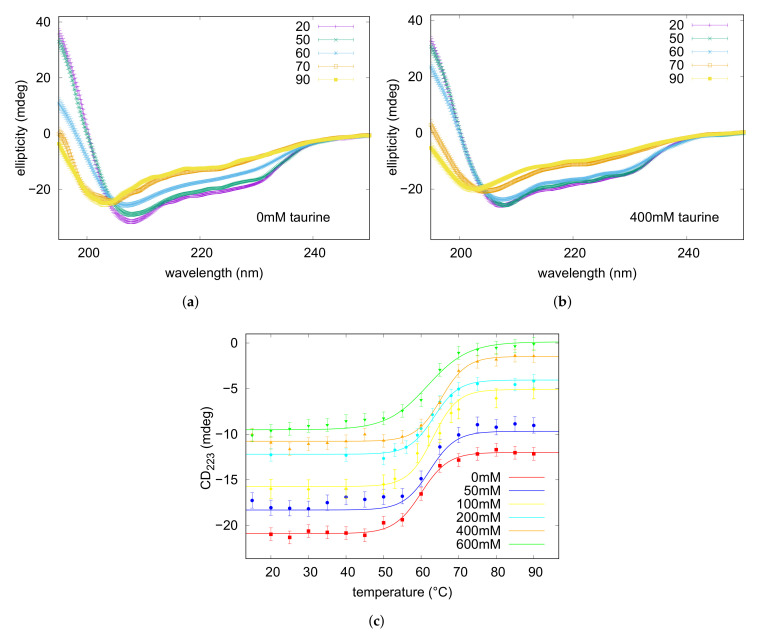
Representative CD spectra recorded at variable temperatures on lysozyme solutions in the absence (**a**) and in the presence of taurine 400 mM (**b**). Temperatures corresponding to each trace are reported in the upper legends expressed in ${}^{\circ }$ C. In panel (**c**) the denaturing transition curves obtained by the ellipticity values at 222 nm plotted versus temperature are shown. The traces are referred to the lysozyme solution in the absence or in the presence of increasing amounts of taurine as indicated in the legend. Curves are shifted for the sake of clarity. Continuous lines are the result of a theoretical fitting described in Section 3.2.

**Figure 3:**
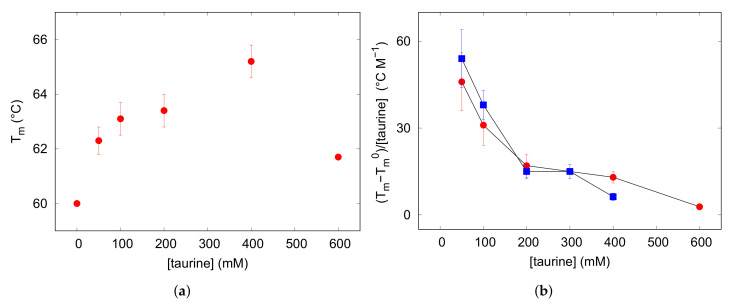
(**a**) panel: Melting temperatures T ${}_{m}$ resulting from CD data obtained from lysozyme acidic solutions, reported as a function of taurine content. (**b**) panel: molar increase of the transition temperature $\left[T_{m}^{i}-T_{m}^{0}\right]/\left[taurine\right]$ calculated for the values shown in the left panel for lysozyme (red circles) and from literature data concerning ubiquitin (blue squares) [37], and plotted against taurine concentration [taurine] (see text in Section 3.2 for an explanation).

The far-UV CD spectra were recorded on lysozyme solution (3 g L ${}^{-1}$ like in SAXS experiments), heating in the range 5–90 ${}^{\circ }$ C pre-eminently at 5 ${}^{\circ }$ C intervals: spectra are shown in Figure 2. The initial trace, maintained up to 45 ${}^{\circ }$ C, displays the typical signature of the native state dominated by the alpha-helix conformation, indeed two minima at 222 and 208 nm are visible beside a positive optical activity below 200 nm. By elevating temperature up to 90 ${}^{\circ }$ C, signal intensities progressively reduced, while a negative band arised at around 202 nm, typical of the random coil state characterising unfolded proteins. The thermal denaturation profile was achieved by plotting the ellipticity values measured at 222 nm, reflecting the helical content ([63,64] and references therein), as a function of temperature (Figure 2). Data were fitted on the basis of a two-state mechanism to a sigmoidal function typical of a highly cooperative unfolding reaction, and a midpoint transition temperature $T_{m}$ of 60 ${}^{\circ }$ C was estimated (see Table 1). For evaluating the protecting effect of taurine against thermal denaturation of the protein, similar heating experiments were performed on lysozyme solutions containing increasing amounts of the osmolyte in the range 50–600 mM. In each case a progressive variation of CD band-shape and intensities, analogous to those monitored for the pure lysozyme solution, were observed with temperature (see Figure 2). Data analysis gave the sigmoidal denaturing transition curves shown in Figure 2, and the obtained $T_{m}$ values are reported in Table 1. It was worthily to notice that data evidenced for the melting temperatures a non-linear—i.e., parabolic—dependence on taurine concentration (Figure 3). Indeed, addition of taurine 50 mM produced a $T_{m}$ increase of about 2 ${}^{\circ }$ C, while further taurine boosts until 400 mM raised the $T_{m}$ value to ≃65 ${}^{\circ }$ C. However, in the presence of taurine 600 mM $T_{m}$ unexpectedly lowered from 65 to 62 ${}^{\circ }$ C. This behaviour observed by varying taurine concentration [taurine] may be rationalized by expressing the results as the molar increase of the transition temperature, $\Delta T_{m}/\left[taurine\right]$ [36], as a function of [taurine] (Figure 3): $\Delta T_{m}$ represents the difference between the midpoint transition temperature registered with a certain taurine content ( $T_{m_{i}}$ ) and that one measured in the absence of taurine ( $T_{m}^{0}$ ). As shown in Figure 3, red circles in (b) panel, a progressive decrease of this parameter can be appreciated by growing taurine content suggesting an agreement with an exponential decay extrapolation model. This behavior strictly resembles the one that can be deduced by literature data on ubiquitin [37] (blue squares in Figure 3b). Ubiquitin is smaller (8.6 kDa) than lysozyme (14.3 kDa), and their isoelectric points are quite different, too (pI = 11.2 for lysozyme and pI = 6.8 for ubiquitin). The different, but similar tendency of $\Delta T_{m}/\left[taurine\right]$ as a function of [taurine] in solution, suggests that the osmolyte protective and stabilizing action could be related to mixtures properties and not to single protein peculiarities. An action contrasting the stabilizing effect of taurine was noticed in the presence of ionic strength: addition of NaCl 150 mM to lysozyme in the presence of taurine 400 mM lowered $T_{m}$ from 65 to ca. 62 ${}^{\circ }$ C (data not shown), confirming the in vitro annihilation of the protecting effect of osmolites when noticeable ionic strenghts are present in solution [45].

### 3.3. Amyloid Aggregation

**Figure 4:**
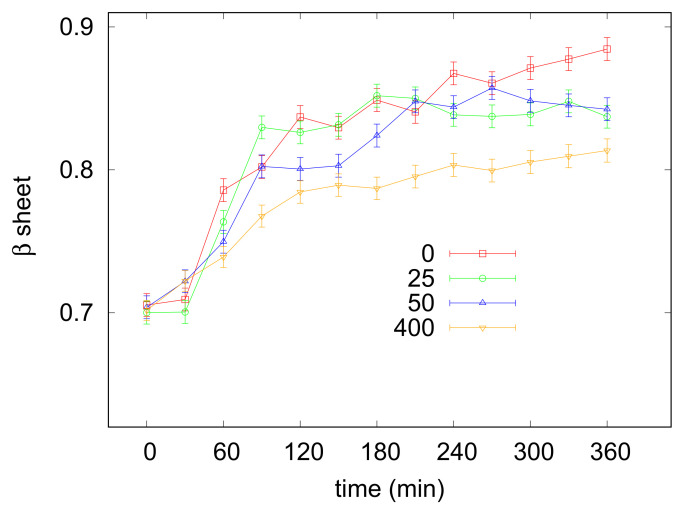
Results of UV/Vis spectrophotometry measurements perfomed during lysozyme fibrillation with CR addition. In the vertical axis, values proportional to β sheet contents are reported, obtained by the ratio $\frac{Abs_{538}}{Abs_{505}}$ measured for lysozyme at acidic conditions and concentration equal to 3 g L ${}^{-1}$ . Taurine content in solution is reported in the legend in mM units. Error bars derive from the average of several experiments, as detailed in Section 2.3.

Lysozyme has been shown to undergo amyloid aggregation under various selected in vitro conditions. Here a specific protocol, operating at 65 ${}^{\circ }$ C and low pH (2.3) was exploited to convert the protein into the highly ordered β -sheeted fibrillar aggregates [44] and the effect of increasing concentrations of taurine (from 25 to 400 mM) in the presence of NaCl 5 mM was investigated. Amyloid fibrillation was monitored with UV/Vis absorption spectroscopy by using CR as a probe for β -sheet formation (see Section 2.3). Lysozyme incubation was at first carried out in the absence of taurine: representative UV spectra recorded at different times after the appropriate treatment with the CR dye are shown in the Supplementary Materials (Figure S2). It can be appreciated that the initial 505 nm peak, due to the free CR, converted with time to a wider signal due to the 538 nm absorption arising from CR bound to fibrils. At each investigated time the ratio between the bound CR and free CR absorption peak intensities was calculated, and collected values, providing the relative amount of β structures, were plotted against the incubation time for evaluating the kinetic of the process. As shown in Figure 4, in the absence of taurine a lag phase of about 30 min was followed by a rapid increase of the aggregation curve reflecting the elongation phase. The plateau indicative of mature fibril formation occurred after the first 150 min. Lysozyme solutions were then incubated, in the same conditions, with increasing amounts of taurine. An exemplificative comparison between UV spectra recorded with or without taurine after 0 and 360 min is reported in in the Supplementary Materials (Figure S3), while the kinetic profiles obtained with taurine 25, 50 and 400 mM are depicted in Figure 4. By considering all profiles just little variabilities are observed during the first 30 min, however it can be seen that increasing concentrations of taurine tended to reduce the elongation rate as well as the final amount of β fibrils formed in solution, as evidenced by the lower level reached by the plateau at saturation. The overall effect was particularly clear for taurine 400 mM (see Table 2). Indeed, in these conditions a reduction of 30% was estimated for the slope of the sigmoid curve, while the plateau level indicative of β -sheet formation was lowered of ca. 40%.

**Figure 5:**
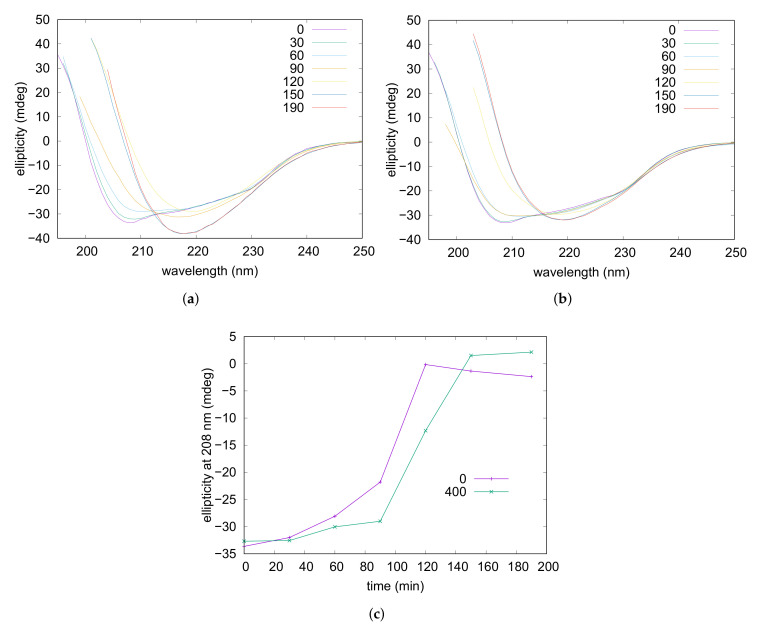
CD spectra recorded at increasing times (expressed in minutes in the legends) of thermal treatment at 65 ${}^{\circ }$ C on lysozyme acidic solutions in the absence (**a**) and in the presence of taurine 400 mM (**b**), and at NaCl 5 mM. Panel (**c**) reports ellipticity values at 208 nm plotted versus time, according to the taurine content reported in the legend in mM units.

Amyloid fibrillation was followed also by CD spectroscopy. Experiments were performed on a lysozyme solution containing NaCl 50 mM and on solutions containing NaCl 5 mM in the presence or the absence of taurine 400 mM. The obtained far-UV CD spectra recorded at different times of incubation are reported in Figure 5 (and in Figure S4) and show that, for all the three samples, the characteristic double minima of the native alpha helix (at 208 and 222 nm) gradually reduced by leaving the place to a deep minimum at 218 nm typical of the β -sheet conformation [62]. By examining the CD traces it can be noticed that ellipticities at 208 nm are negligible when β -sheets are the predominant form in solution, thus monitoring of these values during fibrillation might reflect with good approximation the progressive reduction of the alpha helix content accompanying the process. The obtained curves where the ellipticities at 208 nm were plotted versus the incubation time are shown in Figure 5c. They reveal that addition of taurine resulted in a significant delay of the process, while by increasing the ionic strength—NaCl from 5 mM to 50 mM—the alpha helix unfolding was noticeably anticipated (see Figure S4). In particular, after 90 min of incubation ellipticities reduced of around 78% and 35% in the presence of NaCl 50 and 5 mM respectively, while for the sample containing the osmolyte a reduction of only 10% was observed. Moreover, after 120 min of incubation in the absence of taurine the signal was almost nullified while in the presence of taurine ≃35% of the initial intensity was still present. With concern to additional structural information, any reliable shift of band position possibly related to structural or morphological diversity of corresponding β -sheet structures [65] could be inferred from comparison of final spectra obtained in the examined conditions. Nevertheless, diminishing of CD intensity at 218 nm evidenced by lowering the ionic strength and even more by addition of taurine (Figure 5) could be ascribed to a progressive reduction in quantity and/or dimensions of the β -sheet assemblies, in accordance to results collected by UV analysis described above.

**Figure 6:**
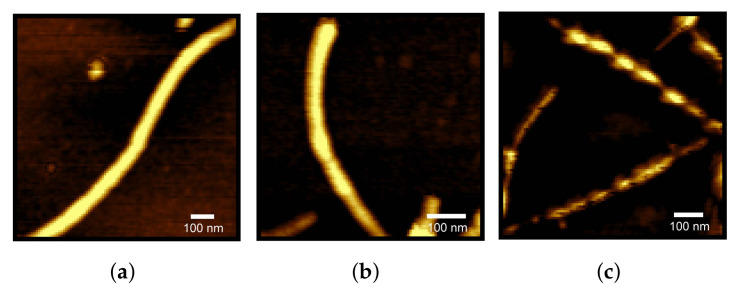
AFM images recorded in Non-contact mode, corresponding to lysozyme fibrillated without taurine (**a**), with taurine 25 mM (**b**) and with taurine 400 mM (**c**).

At last, AFM measurements provided the resulting structures arising from 6 h of fibrillation, shown in Figure 6 and in the Supplementary Materials (Figure S5). Each experimental condition reported the presence of fibrils and protofibrils. Height profiles of fibrils, corresponding to their diameters, are reported in the Supplementary Materials (Figure S6), and they agree with literature data on lysozyme fibrils [66]. In some images (see panel (c) of Figure 6) the fibril pitch appears in a clear way. However, and we could not identify any detectable and statistically meaningful pitch difference between fibrils obtained at different taurine content. On the other side, some aggregates of globular shape appeared at increasing taurine content (see Figure S5), although no Maltese crosses were observed on samples with polarized light microscope, suggesting they were not spherulites [67].

**Figure 7:**
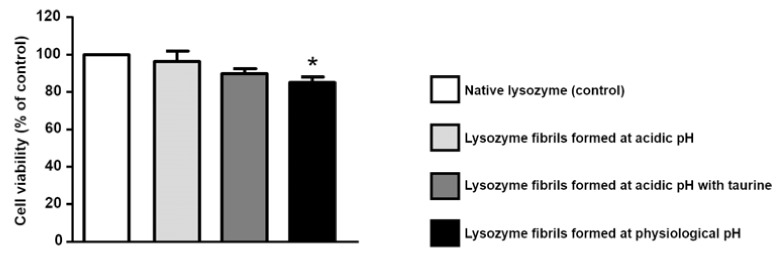
Effect of lysozyme fibrils on the survival of SH-SY5Y neuroblastoma cells. Lysozyme fibrils were added at a concentation of 20 μ M, in the presence or absence of taurine (25 mM). After 72 h, cell viability was assessed by the MTT reduction assay. MTT reduction was expressed as a percentage of the control (native lysozyme). Statistical differences were assessed by one-way ANOVA followed by Dunnett’s post hoc test. F (4, 20) = 5.591. Each column represents the mean ± S.E.M. of *n* = 4–6 experiments performed in triplicate. * Significant versus CTL (*p* < 0.05).

In light of the fact that taurine is able to increase lysozyme thermal stability, and to one-sidedly modify its amyloid aggregation pattern, and in consideration of the fact that its use against AD has gained positive results [28], we performed a cytotoxicity investigation on lysozyme aggregation kinetic final products, obtained in absence or in presence of taurine. The cytotoxicity of lysozyme aggregates was measured by studying their effect at a total protein concentrations of 20 μ M. As shown in Figure 7, cell viability was not affected neither by native lysozyme nor by the exposure to lysozyme fibrils formed at acidic pH. Fibrils obtained at acidic pH in the presence of 400 μ M taurine were also tested. As reported in the same graph, the presence of taurine did not modify cell viability. Conversely, when SH-SY5Y cells were exposed to lysozyme fibrils formed at physiological pH, we observed a significant reduction in cell viability, in agreement with literature results [53]. Of note, the exposure to native lysozyme in the presence of taurine induced a slight but significant reduction in cell survival (data not shown), even though the exposure to lysozyme fibrils formed at acidic pH in the presence of taurine did not affect cell viability. Further experiments are needed to establish the effect of taurine in these different experimental settings.

## 4. Discussion and Conclusions

In the present study we report on the ability of taurine to modify lysozyme stability, and to interphere with its amyloid aggregation propensity. Also, we explored taurine influence on the cytotoxicity of the resulting fibrils.

Nowadays there is a growing awareness of the health benefits of taurine including calcium homeostasis, prevention of obesity, recovery from osmotic shock, protection against glutamate excytotoxicity, prevention of epileptic seizures, and anti-neurotoxic and anti-inflammatory effects are supposed as well [32]. These assumed benefits determined an increase in the global taurine market size, which was USD 264 million in 2020 and is expected to reach USD 434 million in 2021 [68]. Nevertheless, contrary to other osmolytes present in cells like trimethylamine N-oxide [17,69,70,71,72], taurine had not been widely investigated in its molecular interactions with biomolecules, neither in its modification of water network until now, with the exception of a few recent works [37,59,73], at our knowledge. Thus, to fill the gap concerning this osmolyte represents an important challenge, above all for its relevance in several medical issues and its increasing consumption. Our work started from the evaluation of taurine effects on lysozyme interactions when the protein was in solution in its stable native state. SAXS experiments proved that even low amounts of taurine modified protein-protein interactions: the short-range attractive term decreases, as well as the long-range electrostatic repulsion between proteins decreases because the dielectric permittivity of the solvent increases at increasing taurine content. The short-range attractive term reduction can be related to changes due to the presence of different hydration contribution [74]. On the other hand, no change in protein solvation shell could be detected by SAXS data. Melting experiments showed that taurine increased the T ${}_{m}$ value associated to lysozyme thermal denaturation, and as seen for other osmolytes effectiveness depended on taurine concentration. By confirming data previously reported [39], lysozyme $T_{m}$ positively correlated with taurine molarity up to 400 mM, however a further increase of the osmolyte content produced a decrease of the transition midpoint. As a result, a parabolic trend of $T_{m}$ vs. taurine concentration was detected. In this context, it was interesting to express the osmolyte protecting effect as the molar increase of $T_{m}$ ( $\left(T_{m}-T_{m}^{0}\right)/\left[taurine\right]$ ), as an almost exponential decrease of this value vs taurine concentration emerged. Even more, it was peculiar to find a comparable trend for the same parameter in the case of a taurine-ubiquitin system, previously studied by Brudziak et al. (see Figure 3b) [37]. This property, likely related also to the features of the protein and/or to external factors (e.g., pH, ionic strength), appears to be a good instrument that may help in shedding light on the osmolyte-water-protein system properties, as it may assist in monitoring factors conditioning the stabilizing or destabilizing power. The taurine stabilizing effect has been recently ascribed to the interaction of the $NH_{3}^{+}$ group—able to enhance the water network—with protein surface, whereas the $SO_{3}^{-}$ group considered as a weakening of the protein hydration shell is oriented far away (see [35]). Moreover, a limited number of interaction centres are supposed to exist on protein surface, and with growing of osmolyte concentration weakly interacting molecules gradually increase in solution up to overcome the number of stabilizing molecules. In agreement with the work of Brudziak et al. [37], our results could be explained by assuming that weakly interacting species, lacking of a specific orientation, cannot avoid the $SO_{3}^{-}$ destabilizing interaction with the protein surface and, as a result, the protecting action of taurine normalized for its molarity is seen to progressively decrease.

A good parallel did emerge between the osmolyte inhibiting effects observed on lysozyme thermal denaturation and amyloid fibrillation explored by UV/Vis measurements for taurine ≤400 mM. Presumably due to the stressful conditions here adopted (stirring and pH 2.3), strongly accelerating the process, a clear evaluation of the lag phase in the different conditions could not be derived, however with concern to the elongation rate it was seen that gradually decreased with taurine molarity, as well as the final amount of β -sheet structures revealed by CR interaction.

These latter data indicate that taurine could inhibit the side-by-side and/or head-to-head interactions between protofibrils dominating the elongation phase. On the other hand, our melting experiments highlighted taurine ability in stabilizing lysozyme in its folded native state, and it is known that fibrillogenesis, and nucleation in particular, require unfolded or misfolded conformations to take place. On these bases, our study suggests that taurine might also reduce fibril formation by contrasting the primary and secondary nucleation processes, being the effect proportional to its concentration at least up to 400 mM. In this framework, also the property depicted by SAXS analysis could be considered. In particular, with concern to the inhibiting effect exerted on short-range intermolecular protein interactions, a possible role in the early stage of amyloid fibrillation cannot be excluded. UV/Vis results collected on amyloid aggregation experiments were corroborated by CD spectroscopy, too. Spectra recorded on fibrillated samples showed in the presence of taurine a less intense signal intensity, in possible agreement with lower amounts of β -sheet structures. Moreover, as shown by profiles describing the reduction of the alpha helix content, CD data evidenced, also at the molecular level, a significant delaying effect exerted by taurine. AFM images showed that, either in absence or in presence of osmolyte, fibrillar structures were formed, although analogous morphologies were detected in the different examined cases. On the other hand, preliminary investigation performed on cell viability did not evidence a substantial variability related to the presence of the osmolyte. In view of these results we consider that the evaluation of the inhibiting effects of biological or synthetic compounds on protein amyloidogenesis could not let off a cytotoxicity analysis of the products of aggregation. Although it is well known that the protein ensemble resulting from an amyloid aggregation pattern is generally quite assorted, ranging from misfolded monomers to oligomers of different size, until protofibrils and fibrils, and that every element may possess different cytotoxicity, inhibition studies are not always related to cellular investigation. We determined an effect of taurine on lysozyme amyloidogenesis, but by our data it does not impact on aggregates cytotoxicity. When further similar studies on taurine effects on amyloid β peptide and tau aggregations will be performed, we believe that taurine role in AD should become clearer.

## Figures and Tables

**Table 1:** Melting temperature obtained from CD data analysis.

[taurine] (mM)	$T_{m}$ ( ${}^{\circ }$ C)
0	60.0 ± 0.1
50	62.3 ± 0.5
100	63.1 ± 0.6
200	63.4 ± 0.6
400	65.2 ± 0.6
600	61.7 ± 0.1

**Table 2:** Ratios between absorbances detected at 538 and 505 nm during lysozyme fibrillation at two precise time points: after 180 and 360 min from the beginning of the process. Experiments were performed at increasing taurine contents, as reported in the first column of the table.

[taurine]	$\frac{{\mathrm{Abs}}_{538}}{{\mathrm{Abs}}_{505}}$	$\frac{{\mathrm{Abs}}_{538}}{{\mathrm{Abs}}_{505}}$
(mM)	after 180 min	after 360 min
0	0.84 ± 0.01	0.88 ± 0.01
25	0.85 ± 0.01	0.83 ± 0.01
50	0.82 ± 0.01	0.84 ± 0.01
400	0.78 ± 0.01	0.81 ± 0.01

## Supplementary Materials

The following supporting information can be downloaded at: https://www.mdpi.com/2075-1729/12/1/133/s1, Figure S1: SAXS curves corresponding to lysozyme at acidic pH in absence of taurine at concentration equal to 10 (violet) and 3 (green) g L ${}^{-1}$ : a structure factor due to protein-protein interactions is evidenced by increasing protein concentration. Figure S2. UV spectra recorded, after CR addition, on a 3 g L ${}^{-1}$ lysozyme solution at pH 2.3, 65 ℃, in agitation during the fibrillation experiment. Figure S3. Absorption curves of lysozyme 3 g L ${}^{-1}$ in presence of Congo Red at the beginning and at the end of the fibrillation processes. Figure S4. Left: CD spectra recorded at increasing times (expressed in minutes in the legend) of thermal treatment at 65 °C on lysozyme acidic solutions with NaCl 50 mM in the absence of taurine 400 mM. Figure S5. AFM images: Lysozyme after fibrillation without taurine (A) and 25 mM (B), 400 mM of taurine (C). Figure S6. Height profiles derived from AFM image.

## Abbreviations

The following abbreviations are used in this manuscript:

MDPI	Multidisciplinary Digital Publishing Institute
SAXS	Small Angle X-ray Scattering
CD	circular dichroism
