# Supplementary material for: Taurine Stabilizing Effect on Lysozyme
Source: Life (Basel). 2022 Jan 17;12(1):133. doi: 10.3390/life12010133 (PMC8779517; doi:10.3390/life12010133)
Supplement: Supplementary file 1 [file life-12-00133-s001.zip › life-1530329-supplementary.pdf]

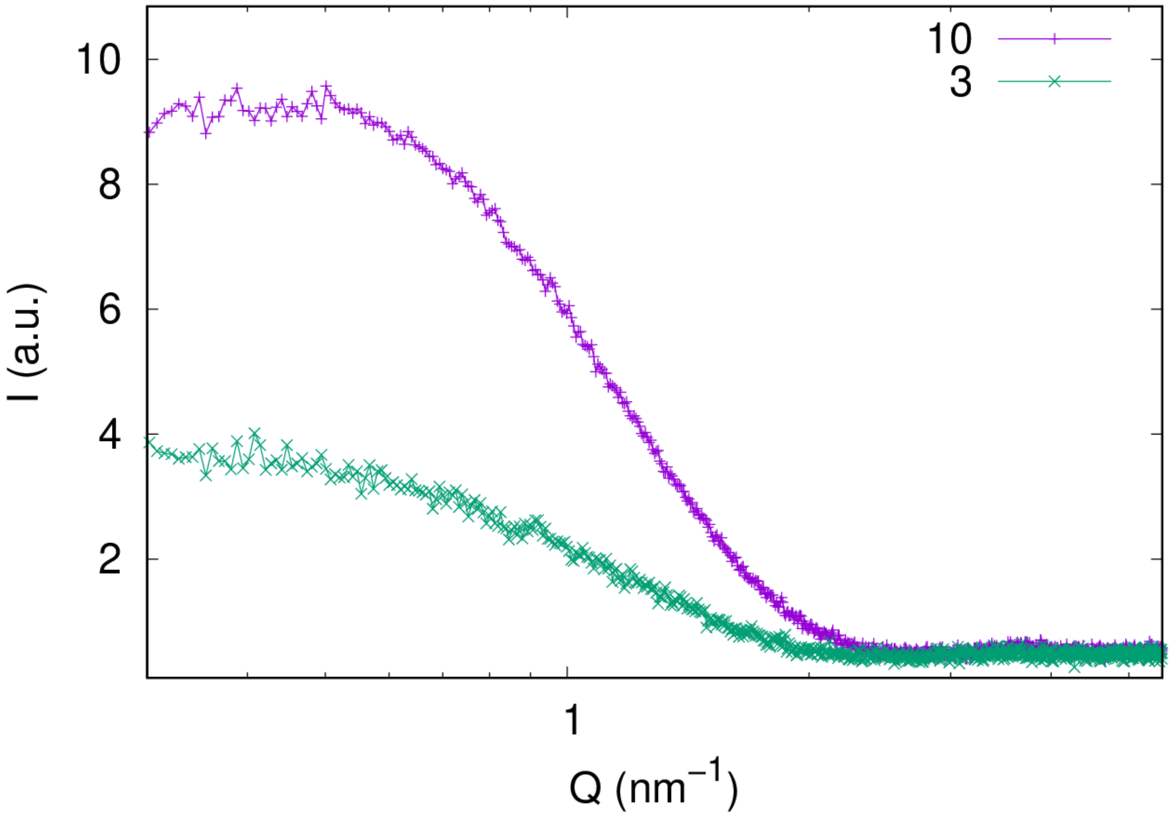

Figure S1: SAXS curves corresponding to lysozyme at acidic pH in absence of taurine at concentration equal to 10 (violet) and 3 (green) g L^{-1}: a structure factor due to protein-protein interactions is evidenced by increasing protein concentration.

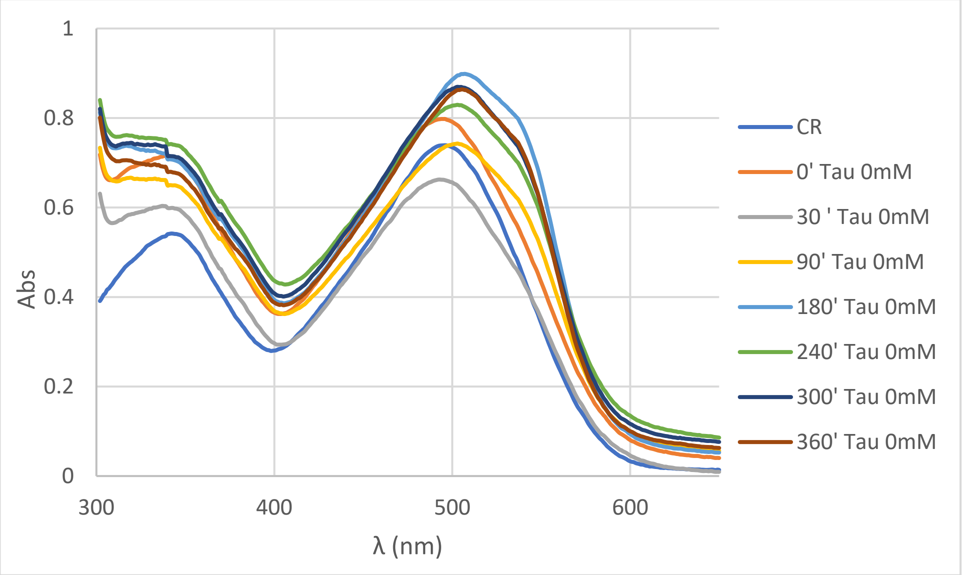

Figure S2. UV spectra recorded, after CR addition, on a 3 g L^{-1} lysozyme solution at pH 2.3, 65 ℃, in agitation during the fibrillation experiment: the different incubation times are indicated in the legend.

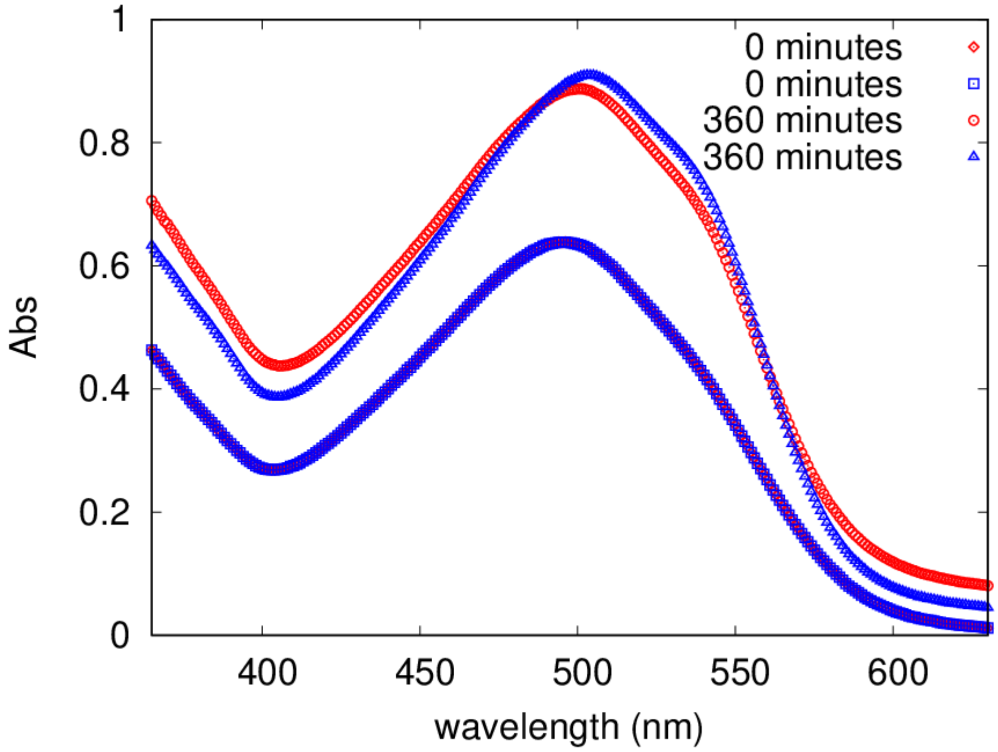

Figure S3. Absorption curves of lysozyme 3 g L^{-1} in presence of Congo Red at the beginning and at the end of the fibrillation processes. Legend reports the investigated time steps. Red colour refers to lysozyme solution in absence of taurine. Blue colour refers to lysozyme solution with 400mM taurine. Curves corresponding to the beginning of the process completely overlap.

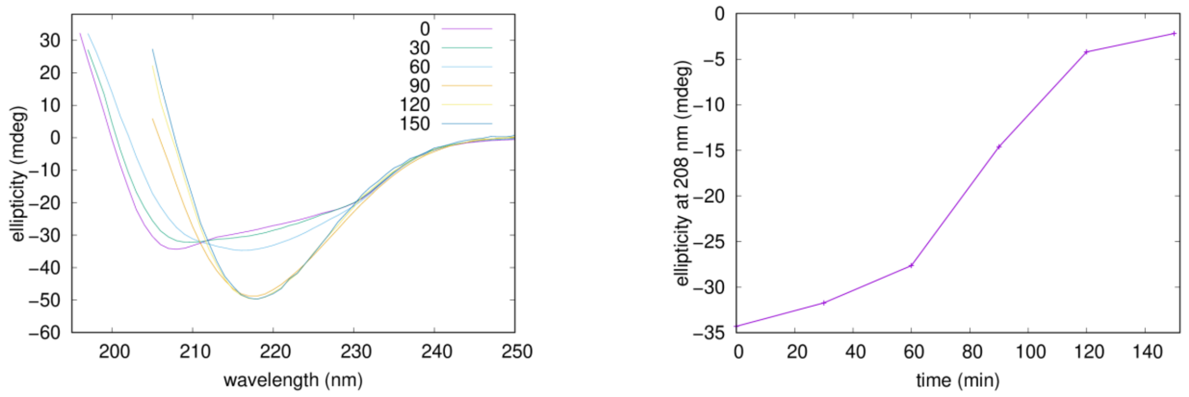

Figure S4. Left: CD spectra recorded at increasing times (expressed in minutes in the legend) of thermal treatment at 65°C on lysozyme acidic solutions with NaCl 50mM in the absence of taurine 400mM. Right: ellipticity values corresponding to the same sample conditions at 208 nm plotted versus time.

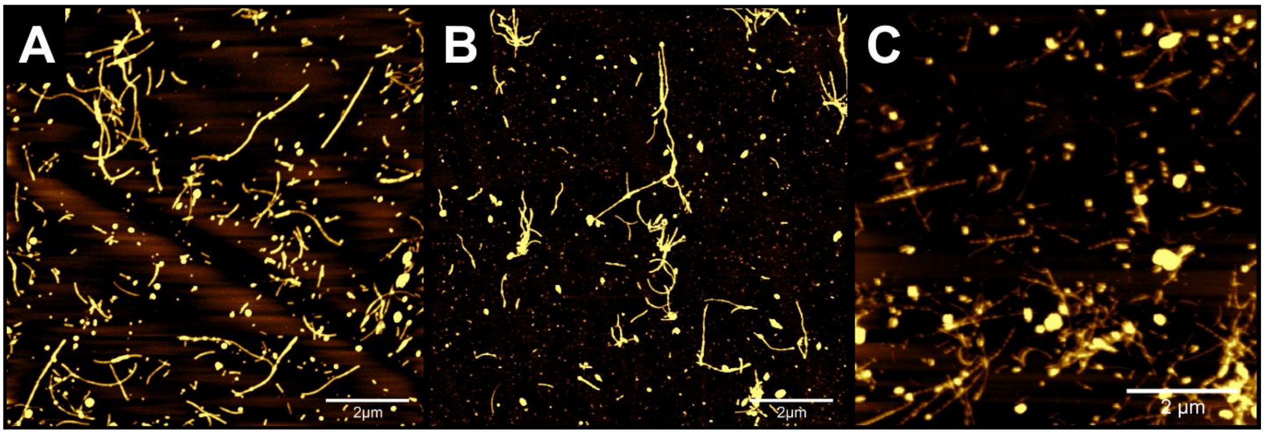

Figure S5. AFM images: Lysozyme after fibrillation without taurine (A) and 25 mM (B), 400 mM of taurine (C).

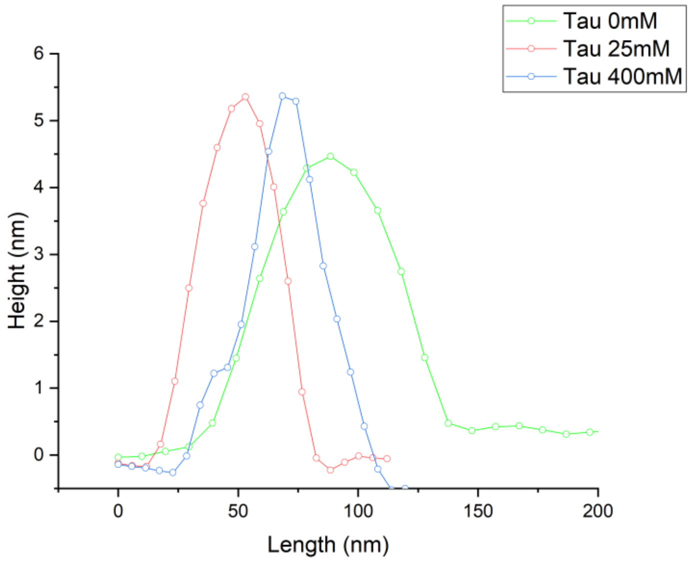

Figure S6. Height profiles derived from AFM image. Height profiles of all fibrils are compatible with literature (see Reference [66] in the manuscript).
